# Chronic intermittent hypoxia increases encoding pigment epithelium-derived factor gene expression, although not that of the protein itself, in the temporal cortex of rats*,**

**DOI:** 10.1590/S1806-37132015000100006

**Published:** 2015

**Authors:** Guilherme Silva Julian, Renato Watanabe de Oliveira, Vanessa Manchim Favaro, Maria Gabriela Menezes de Oliveira, Juliana Cini Perry, Sergio Tufik, Jair Ribeiro Chagas

**Affiliations:** Federal University of São Paulo, Paulista School of Medicine, Department of Psychobiology, São Paulo, Brazil. Department of Psychobiology, Federal University of São Paulo Paulista School of Medicine, São Paulo, Brazil; Federal University of São Paulo, Paulista School of Medicine, Department of Psychobiology, São Paulo, Brazil. Department of Psychobiology, Federal University of São Paulo Paulista School of Medicine, São Paulo, Brazil; Federal University of São Paulo, Paulista School of Medicine, Department of Psychobiology, São Paulo, Brazil. Department of Psychobiology, Federal University of São Paulo Paulista School of Medicine, São Paulo, Brazil; Federal University of São Paulo, Paulista School of Medicine, Department of Psychobiology, São Paulo, Brazil. Department of Psychobiology, Federal University of São Paulo Paulista School of Medicine, São Paulo, Brazil; Federal University of São Paulo, Paulista School of Medicine, Department of Psychobiology, São Paulo, Brazil. Department of Psychobiology, Federal University of São Paulo Paulista School of Medicine, São Paulo, Brazil; Federal University of São Paulo, Paulista School of Medicine, Department of Psychobiology, São Paulo, Brazil. Department of Psychobiology, Federal University of São Paulo Paulista School of Medicine, São Paulo, Brazil; Federal University of São Paulo, Department of Biosciences, Santos, Brazil. Department of Psychobiology, Federal University of São Paulo Paulista School of Medicine, São Paulo; and Department of Biosciences, Federal University of São Paulo, Baixada Santista Campus, Santos, Brazil

**Keywords:** Sleep apnea, central, Disease models, animal, Cognition, Sleep, Sleep apnea, obstructive

## Abstract

**Objective:**

Obstructive sleep apnea syndrome is mainly characterized by intermittent hypoxia (IH) during sleep, being associated with several complications. Exposure to IH is the most widely used animal model of sleep apnea, short-term IH exposure resulting in cognitive and neuronal impairment. Pigment epithelium-derived factor (PEDF) is a hypoxia-sensitive factor acting as a neurotrophic, neuroprotective, and antiangiogenic agent. Our study analyzed performance on learning and cognitive tasks, as well as *PEDF* gene expression and PEDF protein expression in specific brain structures, in rats exposed to long-term IH.

**Methods:**

Male Wistar rats were exposed to IH (oxygen concentrations of 21-5%) for 6 weeks-the chronic IH (CIH) group-or normoxia for 6 weeks-the control group. After CIH exposure, a group of rats were allowed to recover under normoxic conditions for 2 weeks (the CIH+N group). All rats underwent the Morris water maze test for learning and memory, *PEDF* gene expression and PEDF protein expression in the hippocampus, frontal cortex, and temporal cortex being subsequently assessed.

**Results:**

The CIH and CIH+N groups showed increased *PEDF* gene expression in the temporal cortex, PEDF protein expression remaining unaltered. *PEDF* gene expression and PEDF protein expression remained unaltered in the frontal cortex and hippocampus. Long-term exposure to IH did not affect cognitive function.

**Conclusions:**

Long-term exposure to IH selectively increases *PEDF* gene expression at the transcriptional level, although only in the temporal cortex. This increase is probably a protective mechanism against IH-induced injury.

## Abstract (pt)

**Objetivo:**

A síndrome da apneia obstrutiva do sono caracteriza-se principalmente por episódios de hipóxia intermitente (HI) durante o sono e associa-se a diversas complicações. A exposição à HI é o mais usado modelo animal de apneia do sono, e protocolos de curta duração causam diversos prejuízos cognitivos e neuronais. *Pigment epithelium-derived factor* (PEDF, fator derivado do epitélio pigmentado) é um fator neurotrófico, neuroprotetor e antiangiogênico sensível à hipóxia celular. Nosso estudo analisou o desempenho em tarefas cognitivas e de aprendizagem, bem como a expressão do gene *PEDF* e da proteína PEDF em estruturas cerebrais específicas em ratos expostos a HI de longa duração.

**Métodos:**

Ratos Wistar foram expostos a HI (21-5% de oxigênio) durante 6 semanas - o grupo HI crônica (HIC) - ou a normóxia durante 6 semanas - o grupo controle. Após a exposição à HIC, um grupo de ratos foi exposto a normóxia durante 2 semanas (o grupo HIC+N). Todos os animais foram submetidos ao labirinto aquático de Morris para avaliação de memória e aprendizado; avaliou-se também a expressão do gene *PEDF* e da proteína PEDF no hipocampo e nos córtices frontal e temporal.

**Resultados:**

Os grupos HIC e HIC+N apresentaram um aumento de expressão do gene *PEDF* no córtex temporal, porém sem aumento dos níveis proteicos. A expressão do gene *PEDF* e da proteína PEDF manteve-se inalterada nas demais estruturas. A exposição de longa duração à HI não afetou a função cognitiva.

**Conclusões:**

A exposição de longa duração à HI aumenta seletivamente a expressão do gene *PEDF* ao nível transcricional, embora apenas no córtex temporal. Esse aumento é provavelmente um mecanismo de proteção contra a HI.

## Introduction

Obstructive sleep apnea (OSA) is the most common sleep-related breathing disorder and is a public health issue because of its high prevalence. ^(^ 1 ^,^ 2 ^)^ It is characterized by recurrent episodes of partial or complete upper airway obstruction, leading to sleep fragmentation, hypercapnia, and nocturnal intermittent hypoxia (IH). A number of animal models of OSA have been developed over the years,^(^ 3 ^)^ most of which have focused on IH. The IH model produces several effects that are similar to those of OSA, including cognitive impairment, changes in sleep architecture, insulin resistance, and hypertension. ^(^ 4 ^-^ 10 ^)^ This suggests that IH plays an important role in OSA,^(^ 11 ^)^ affecting even cognition. 

Learning and cognition require a process known as synaptic plasticity, which is the ability of synapses to strengthen or weaken their connections.^(^ 12 ^)^ Several factors control synaptic plasticity, including neurotransmitters and neurotrophic factors that play an essential role in the growth and survival of developing neurons. One such factor is pigment epithelium-derived factor (PEDF), which has antiangiogenic, neuroprotective, and neurotrophic activity. 

As a neuroprotective agent, PEDF reduces glutamate-mediated excitotoxicity^(^ 13 ^-^ 15 ^)^ and attenuates ischemic brain damage.^(^ 16 ^)^ As a neurotrophic agent, PEDF induces the expression of other factors, such as brain-derived neurotrophic factor, glial cell line-derived neurotrophic factor, and nerve growth factor,^(^ 17 ^)^ and increases the formation of dendritic spines. ^(^ 18 ^)^ The sensitivity of PEDF to hypoxic exposure has been shown to vary,^(^ 19 ^,^ 20 ^)^ with divergent results regarding the relationship between PEDF and oxygen levels. 

Although the effects of short-term IH on learning, cognition, memory, and neurotrophic factors are known,^(^ 21 ^)^ the effects of long-term IH remain unclear. Therefore, in order to determine the relationships among PEDF, chronic intermittent hypoxia (CIH), and memory, as well as to improve the understanding of the role of PEDF in CIH, the present study examined spatial memory, *PEDF* gene expression, and PEDF protein expression in a rat model of CIH. Indeed, the effects of in vivo hypoxia models on PEDF messenger RNA (mRNA) and protein levels remain unknown. 

## Methods

In the present study, we used 45 adult male Wistar Hannover rats provided by the Federal University of São Paulo *Centro de Desenvolvimento de Modelos Experimentais para Medicina e Biologia* (CEDEME, Center for the Development of Biological and Biomedical Models), located in the city of São Paulo, Brazil. The study was approved by the Animal Research Ethics Committee of the Federal University of São Paulo, located in the city of São Paulo, Brazil (Protocol nº 2025/11). 

All animals were housed at 22ºC on a 12/12-h light/dark cycle (lights on at 7:00 a.m. and off at 7:00 p.m.) and were given *ad libitum* access to food and water. The rats were randomly assigned to the control group (n = 15); the CIH group (n = 15), which comprised animals exposed to IH for 6 weeks^(^ 22 ^)^; or the CIH+N group, which comprised animals exposed to 6 weeks of IH followed by 2 weeks of recovery in normoxia.^(^ 22 ^)^

The Morris water maze (MWM) test was performed in a separate room in a black circular pool (of 200 cm in diameter by 40 cm in height) filled with water at approximately 23ºC to a depth of 25 cm. For animal orientation, distinct visual cues were placed on each wall of the room. A black platform of 10 cm in diameter was placed 2 cm below the surface and fixed in the center of the target quadrant. 

Two separate tests were performed. The first involved 8 control rats and the 15 rats in the CIH group, and the second involved 7 control rats and the 15 rats in the CIH+N group. Spatial learning sessions were conducted on five consecutive days, in the last week of exposure to CIH or CIH+N. During the test week, the rats in the CIH group were exposed to IH for 8 h/day. The spatial learning sessions consisted of four 1-min trials for each animal, with a 1-min interval between trials. Rats began the MWM from different quadrants in the pool at the start of each trial. 

After the last training day, all rats underwent a 1-min trial of free swimming in the MWM without the platform. The ratio between the time spent in the target quadrant and the time spent in other quadrants was used in order to determine spatial memory. The tests were performed one day after the last CIH exposure for the CIH group and after the 2 weeks of normoxia for the CIH+N group. Each group had its own control group. All trials were analyzed by identifying contrast between the (white) animal and the (black) tank, with the Noldus EthoVision XT video tracking software, version 7.0 (Noldus Information Technology Inc., Leesburg, VA, USA). 

Immediately after the MWM test, all rats were euthanized by rapid decapitation. The brains were rapidly removed and dissected to remove the hippocampus, frontal cortex, and temporal cortex. All tissues were rapidly dissected on dry ice and stored at −80ºC until RNA extraction. It has been reported that CIH affects spatial memory and learning,^(^ 6 ^,^ 9 ^,^ 23 ^)^ which is why we studied PEDF expression in the hippocampus, frontal cortex, and temporal cortex (areas related to spatial and visual learning). 

For all structures, total RNA extraction was performed with TRIzol^(r)^ (Thermo Fisher Scientific Inc., Waltham, MA, USA), in accordance with the manufacturer instructions. After extraction, RNA was treated with DNAse I (Thermo Fisher Scientific Inc.), its quality and integrity being evaluated by visualization of rRNA after agarose gel electrophoresis. Quantitation was performed by means of spectrophotometry at 260 nm (NanoDrop, Wilmington, DE, USA), and purity was estimated by a 260/280-nm ratio > 1.8. One µg of RNA from each dissected structure was reverse transcribed with the High Capacity cDNA Reverse Transcription Kit (Applied Biosystems, Foster City, CA, USA), in accordance with the manufacturer instructions. 

Each cDNA was used as a template for real-time PCR amplification with fluorescent-labeled probes (TaqMan^(r)^; Applied Biosystems) and the 7500 Real-Time PCR System (Applied Biosystems) for detection. The level of expression of the *PEDF* gene (Rn00709999_m1) was determined by using *beta-actin* (Rn00667869_m1) and *glyceraldehyde-3-phosphate dehydrogenase* (Rn01775763_g1) as housekeeping genes.^(^ 22 ^)^ Each reaction was performed in a final volume of 20 µL, i.e., 1 µL of cDNA diluted in water and 19 µL of master mix (1 µL of TaqMan^(r)^ assay probe, 10 µL of TaqMan^(r)^ Universal PCR Master Mix, and 8 µL of water), threshold cycle values being maintained between 15.0 and 33.0. All samples were run in triplicate, and average values were calculated. 

For Western blotting analyses, all tissues were homogenized in lysis buffer (50 mM Tris-HCl, pH 7.4; 100 mM NaCl; 0.1% Triton X-100 [The Dow Chemical Company, Midland, MI, USA]; 1 mM EDTA; and a protease inhibitor cocktail [Sigma-Aldrich, St. Louis, MO, USA]), 10 µL of lysis buffer being used for each 1 mg of tissue. After homogenization, the lysate was cleared by centrifugation at 13,000 rpm for 10 min at 4ºC, the supernatant was collected, and supernatant proteins were quantified by the Lowry method (Bio-Rad Laboratories, Inc., Hercules, CA, USA). 

One hundred micrograms of protein extract from all brain structures were incubated at 95ºC for 10 min with sample buffer, subjected to SDS-PAGE (10%), and transferred to a 0.2-µm nitrocellulose membrane (Hybond ECL; GE Healthcare, Chalfont St Giles, UK). After protein transfer, the membrane was blocked in a solution of 5% skim milk in TBS with Tween 20 for 2 h at room temperature and incubated overnight at 4ºC with PEDF primary antibody (BioProducts MD, LLC, Middletown, MD, USA) and glyceraldehyde-3-phosphate dehydrogenase primary antibody (Sigma-Aldrich) at 1:500 and 1:1,000,000 dilutions, respectively. The membrane was developed with goat anti-rabbit secondary antibody, labeled with Alexa Fluor^(r)^ 680 fluorescent dye (Thermo Fisher Scientific Inc.), incubated for 1 h in blocking buffer at 1:10,000 dilution, washed with TBS with Tween 20, and scanned on an Odyssey Infrared Imaging System (LI-COR Biosciences, Lincoln, NE, USA). The images were analyzed with the Odyssey Application Software, version 1.2 (LI-COR Biosciences). 

All data were initially analyzed for normality of distribution and homogeneity of variance with the Kolmogorov-Smirnov test and Levene's test, respectively. When data were not normally distributed or heterogeneity of variance was identified, a Z score correction was performed, and the groups were compared by one-way ANOVA followed by Dunnett's post hoc test, when necessary. With regard to the Western blotting results, the groups were compared by the Kruskal-Wallis test because of the small number of animals per group. With regard to the MWM test results, the groups were compared by repeated measures ANOVA, followed by Tukey's post hoc test. All data were expressed as mean ± standard error of the mean. The level of significance was set at p ≤ 0.05. 

## Results

**Figure 1 -:**
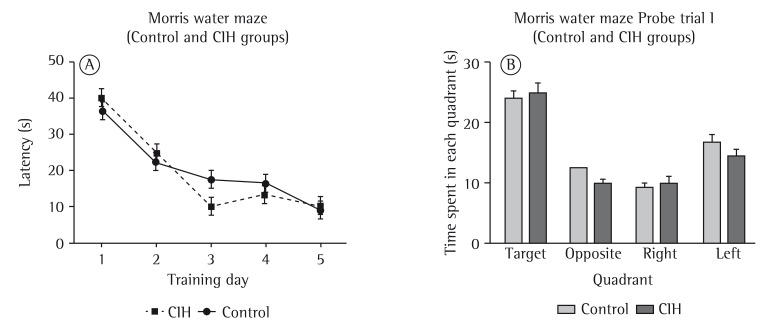
Effects of chronic intermittent hypoxia (CIH) on spatial learning and memory. In A, comparison between Wistar rats exposed to CIH (the CIH group) and control rats (the control group) in terms of their performance during Morris water maze (MWM) testing. For 5 consecutive days, rats underwent four 1-min trials, with a 1-min interval between trials. No statistically significant differences were observed. All data are presented as mean ± SD. Two-way repeated measures ANOVA followed by Tukey's post hoc test. In B, comparison between the control and CIH groups in terms of the time spent in each MWM quadrant, in order to evaluate spatial memory on day 6. No statistically significant differences were observed. All data are expressed as mean ± standard error of the mean.

**Figure 2 -:**
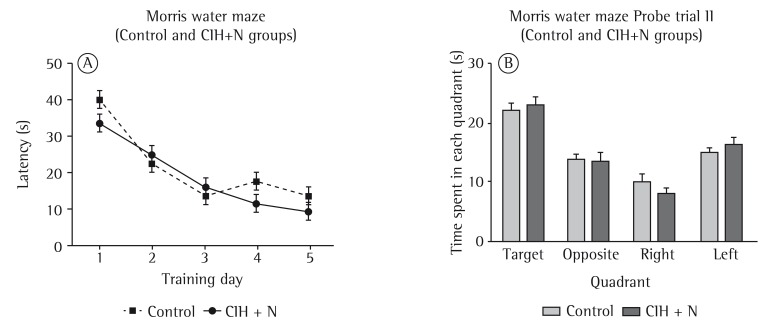
Effects of exposure to chronic intermittent hypoxia (CIH) followed by 2 weeks of normoxia on spatial learning and memory. In A,, comparison between Wistar rats exposed to CIH plus two weeks of normoxia (the CIH+N group) and control rats (the control group) in terms of their performance during Morris water maze (MWM) testing. For 5 consecutive days, rats underwent four 1-min trials, with a 1-min interval between trials. No statistically significant differences were observed. All data are presented as mean ± SD. Two-way repeated measures ANOVA followed by Tukey's post hoc test. In B, comparison between the control and CIH+N groups in terms of the time spent in each MWM quadrant, in order to evaluate spatial memory on day 6. No statistically significant differences were observed. All data are expressed as mean ± standard error of the mean.

All rats learned the MWM, the difference between their performance during the training phase and their performance during the acquisition phase being significantly different. A continuous decrease in latency time shows the learning process (F_1,17_ = 32.561, p < 0.001 for the control group vs. the CIH group during the training phase; F_1,23_ = 38.916, p < 0.001 for the control group vs. the CIH+N group during the training phase). As can be seen in Figures 1 and 2, neither CIH nor CIH+N had any effect on the learning process (F_1,17_ = 1.393, p = 0.246 for the CIH group during the acquisition phase vs. the CIH group during the training phase; F_1,23_ = 1.837, p = 0.128 for the CIH+N group during the acquisition phase vs. the CIH+N group during the training phase), with no evidence of learning impairment in either group when compared with the control group. 

Memory retention can be evaluated by removing the platform (probe trials). A probe trial shows whether animals learned the task and whether they were able to retain learned information. On probe trials I and II, no significant differences were observed between the CIH and control groups or between the CIH+N and control groups in terms of the time spent in the target quadrant (F_1,20_ = 0.122; p = 0.731 and F_1,23_ = 0.278; p = 0.603, respectively; Figures 1 and 2), showing that neither CIH nor CIH+N affected learning and memory retention processes. 

Exposure to CIH did not affect *PEDF* gene expression in the hippocampus and frontal cortex (F_2,21_ = 1.408; p = 0.267 and F_2,21_ = 2.689; p = 0.091, respectively), mRNA levels having remained unaltered after 6 weeks of IH. In addition, *PEDF* gene expression remained unaltered after 6 weeks of IH followed by 2 weeks of recovery in normoxia, showing that although hypoxia induces angiogenesis, CIH and CIH+N did not affect the expression of *PEDF*, which is an important neurotrophic and antiangiogenic factor. 

**Figure 3 -:**
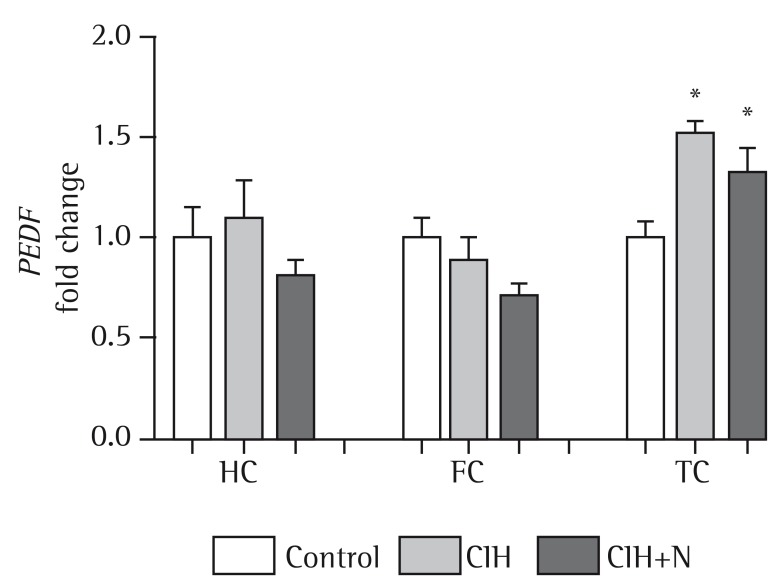
Relative pigment epithelium-derived factor (PEDF) gene expression in the central nervous system, glyceraldehyde-3-phosphate dehydrogenase and betaactin being used as housekeeping genes. PEDF gene expression was found to be increased, although only in the temporal cortex, in the chronic intermittent hypoxia (CIH) and CIH plus 2 weeks of normoxia (CIH+N) groups when compared with the control group. *p < 0.05 in comparison with the control group; one-way ANOVA followed by Dunnett's post hoc test. All data are expressed as mean ± standard error of the mean. HC: hippocampus; FC: frontal cortex; and TC: temporal cortex.

The relative mRNA expression of *PEDF* in the temporal cortex increased 1.5-fold after 6 weeks of exposure to IH (F_2,20_ = 6.583; p = 0.006; Dunnett's post hoc test: p = 0,004). After 2 weeks of recovery in normoxia, *PEDF* mRNA did not return to normal, being 1.3-fold higher in the CIH+N group than in the control group (Dunnet's post hoc test; p = 0.029; Figure 3). This suggests that it takes a long time for the effects of CIH to subside, or even that they are irreversible. 

**Figure 4 -:**
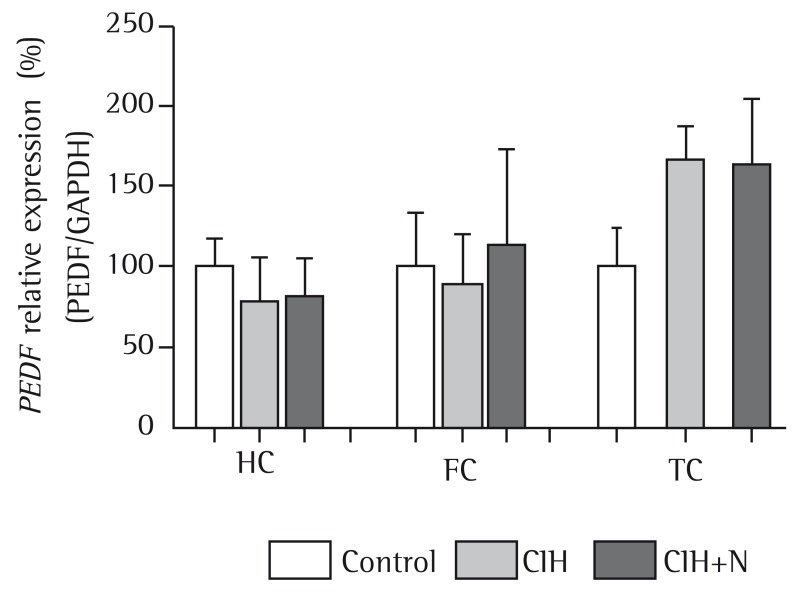
Relative pigment epithelium-derived factor (PEDF) protein expression in the central nervous system, glyceraldehyde-3-phosphate dehydrogenase (GAPDH) being used as loading control. There were no significant differences in PEDF levels among the brain structures studied (p < 0.05). There were no significant differences in PEDF levels among the control, chronic intermittent hypoxia (CIH), and CIH plus 2 weeks of normoxia (CIH+N) groups (p < 0.05). One-way ANOVA. All data are expressed as mean ± standard error of the mean. HC: hippocampus; FC: frontal cortex; and TC: temporal cortex.

There were no significant changes in PEDF protein levels in the hippocampus (H(2) = 1.192; p = 0.551), frontal cortex (H(2) = 0.38; p = 0.981), or temporal cortex (H(2) = 2.577; p = 0.276) of animals exposed to CIH or CIH+N. Although *PEDF *gene expression in the temporal cortex increased 1.5-fold, the protein levels remained unaltered (Figure 4). This might be due to the fact that Western blotting is less sensitive than real-time PCR, which is a much more accurate method. 

## Discussion

Short-term IH models have several neurological implications: increased hippocampal and cortical apoptosis^(^ 9 ^)^; inhibition of cAMP response element binding protein phosphorylation; and learning and memory impairment.^(^ 6 ^)^ Our results show that exposure to CIH and CIH+N did not affect learning and retention in a spatial memory task, being inconsistent with those of studies involving short-term exposure to IH.^(^ 6 ^,^ 9 ^,^ 23 ^-^ 26 ^)^ This might be due to differences in the hypoxia protocol (including differences in cycle and exposure duration) and in oxygen concentration. Studies involving short-term (≤ 30-day) exposure to IH and oxygen concentrations ranging from 21% to 10% have shown evidence of memory impairment (Table 1). A comparison of the findings of the studies described in Table 1 shows that cognitive response varies according to the IH protocol. 

**Table 1 -:** Comparison of different chronic intermittent hypoxia protocols in terms of cognitive effects, evaluation method, minimum oxygen concentration, cycle duration, and exposure duration.

Author	Year of publication	Cognitive effects	Evaluation method	Minimum oxygen concentration	Cycle duration	Exposure duration
Gozal et al.^(9)^	2001	Impaired learning and spatial memory	MWM task	10%	1.5 min	14 days
Row et al.^(25)^	2002	Impaired learning and spatial memory	MWM task	10%	1.5 min	14 days
Goldbart et al.^(6)^	2003	Impaired learning and spatial memory; impaired CREB phosphorylation	MWM task and CREB phosphorylation	10%	1.5 min	1-30 days
Row et al.^(23)^	2007	Impaired working memory	Modified MWM task	10%	1.5 min	1-14 days
Perry et al.^(28)^	2008	Unaltered amygdala-dependent memory	Inhibitory avoidance task	10%	2 min	3-21 days
Wall et al.^(26)^	2013	Reduced LTP	In vitro measurement of LTP	5%	1.5 min	8 days
Shiota et al.^(27)^	2013	Unaltered learning and spatial-memory	MWM task	5%	10 min	8 weeks
Julian et al.^(22)^	-	Unaltered learning and spatial memory	MWM task	5%	3 min	6 weeks and 6 weeks + 2 weeks recovery

MWM: Morris water maze; LTP: long-term potentiation; and CREB: cAMP response element binding protein.

Studies have shown that memory retention processes are not affected by CIH. Shiota et al.^(^ 27 ^)^ demonstrated that exposure to IH for 8 weeks with varying oxygen concentrations (ranging from 21% to 5%) did not affect learning and retention in the MWM task. Golbart et al.^(^ 6 ^)^ evaluated spatial reference memory in rats and demonstrated that short-term exposure to IH selectively affects memory and cAMP response element binding protein phosphorylation, whereas long-term IH exposure does not. Perry et al.^(^ 28 ^)^ reported that rats exposed to IH for 3 weeks did not show impaired acquisition/retention in an inhibitory avoidance task, an amygdala-dependent memory task, or an activity chamber. 

The fact that long-term exposure to IH does not result in memory impairment might be due to neural adaptation after short-term exposure to IH; although short-term exposure to IH affects learning and memory, long-term exposure does not. Therefore, IH models might not be the best animal models to study the cognitive effects of OSA, because of a possible adaptation response in animals.^(^ 29 ^)^

Our study showed that neither CIH nor CIH+N affected *PEDF* gene expression or PEDF protein expression in the hippocampus. These results corroborate our behavioral results, which suggest that the hippocampus was able to exert its spatial learning function normally. Because PEDF has important neurotrophic and neuroprotective functions, PEDF levels are expected to remain unaltered in cases of unaltered learning and memory. 

Our study showed that *PEDF* gene expression in the temporal cortex increased 1.5-fold in the CIH group and 1.3-fold in the CIH+N group. This increase in *PEDF* gene expression is similar to that observed in exposure to severe sustained hypoxia (an oxygen concentration of 0.2%) in vitro; increased *PEDF* gene expression can be a cellular defense mechanism to ensure cell survival under severe hypoxic conditions.^(^ 30 ^)^ In addition, a period of 2 weeks of recovery in normoxia after exposure to CIH is not enough to normalize *PEDF* gene expression, showing that CIH-induced changes can be long-lasting.^(^ 31 ^)^ Although *PEDF* gene expression increased in the CIH and CIH+N groups, PEDF protein levels did not change. Divergent gene and protein expression profiles following severe sustained hypoxia have been reported, protein levels having remained unaltered and genetic expression having increased 2.0-fold.^(^ 30 ^)^

The effects of CIH on *PEDF* gene expression were not reversed after 2 weeks of recovery in normoxia in the CIH+N group. This underscores the fact that CIH has persistent effects on biochemical and oxidative parameters in the brainstem and forebrain, which are related to hypersomnolence.^(^ 31 ^)^

The unaltered protein levels in the present study might also be due to increased PEDF catabolism, given that matrix metalloproteinases 2 and 9 show increased activity in hypoxic conditions and are involved in PEDF degradation. Therefore, increased PEDF levels followed by increased activity of matrix metalloproteinases 2 and 9 might result in unaltered PEDF protein levels.^(^ 32 ^)^ Conversely, VEGF, which is a potent angiogenic factor, is expressed differently among brain regions, VEGF levels remaining unaltered in the temporal cortex and being increased in the frontal cortex after exposure to IH. In addition, VEGF regulates PEDF expression, supporting the idea of a negative feedback loop in the protein.^(^ 33 ^)^

The present study has some limitations. One is that CIH models simulate only one of the four major characteristics of OSA. Another limitation is that our CIH protocol included a low number of IH events per hour (simulating mild OSA). In summary, long-term exposure to IH selectively increased *PEDF* gene expression at the transcriptional level, although only in the temporal cortex. In the hippocampus and frontal cortex, *PEDF* gene expression remained unaltered. Protein expression remained unaltered in all structures. Exposure to CIH did not affect learning and memory on the MWM task. This selective increase in gene expression in the temporal cortex might be a protective mechanism against the neuronal injury caused by CIH. The results of the present study suggest that the effects that long-term exposure to IH has on memory are reversible. 
